# Effects of in-package cold plasma treatment on poultry breast meat packaged in high CO_2_ atmosphere

**DOI:** 10.1016/j.psj.2024.104085

**Published:** 2024-07-10

**Authors:** Hong Zhuang, Michael James Rothrock, Kurt C. Lawrence, Gary R. Gamble, Brian C. Bowker

**Affiliations:** US National Poultry Research Center, USDA-ARS, Athens, GA 30605, USA

**Keywords:** dielectric barrier discharge, campylobacter jejuni, salmonella typhimurium, meat quality, nonthermal plasma

## Abstract

High CO_2_ in packages significantly extends microbiological shelf life of poultry meat. Cold plasma is an emerging antimicrobial treatment, which generates various reactive gas species and inactivates microbials effectively. The objective of this study was to explore the potential effects of combining high CO_2_ package and in-package cold plasma (**IPCP**) treatments on the quality and safety of raw chicken breast meat. Noninoculated samples and samples inoculated with *Campylobacter jejuni* and *Salmonella* Typhimurium were packaged in 0, 30, 70, or 100% CO_2_ (with make-up gas N_2_) and treated with IPCP at 70 kV for 3 min. Ozone formation, microbial counts, drip loss, pH, and color were measured. There was no interaction effect between high CO_2_ package and IPCP on microbial counts, drip loss, and color measurements. IPCP reduced spoilage microbial growth by 0.43 log (from 7.00 log to 6.57 log, *P* = 0.033) and *C. jejuni* populations by 0.67 log (from 4.82 log to 4.15 log, *P* < 0.001) on meat surface but did not affect *S.* Typhimurium (*P* = 0.206). Increased CO_2_ in packages had more effect on spoilage microbial growth (more than 1.5 log from 8.08 log to 6.35 log, *P* < 0.001) and *S.* Typhimurium populations (more than 0.5 log from 4.94 log to 4.39 log, *P* = 0.004) than IPCP but did not affect *C. jejuni* (*P* = 0.163). IPCP resulted in increases in changes in L* by 1.67 units (0.70 vs. 2.37, *P* = 0.016) and a* values by 0.56 units (0.73 vs. 1.29, *P* < 0.001) and decreases in b* values by 0.91 units (0.46 versus −0.45, *P* = 0.015). High CO_2_ levels caused increases in changes in L* values by 4.35 units (−0.82 versus 3.53, *P* < 0.001) with no effects on a* and b* values (*P* > 0.05). Data demonstrate that there are no combined effects by high CO_2_ package and IPCP on meat quality and safety of raw chicken breast meat under our experimental conditions. Either high CO_2_ package or IPCP can retain microbial quality and safety, even though they may cause changes in appearance of stored chicken breast meat.

## INTRODUCTION

Modified atmosphere packaging (**MAP**) can be used for preservation of fresh meat quality. In MAP, O_2_, N_2_, and CO_2_ are the most used components, but CO_2_ is the only one with a direct antimicrobial effect (Arvanitoyannis and Stratakos, 2012). It has been well documented that high CO_2_ in packages can significantly extend microbiological shelf life of poultry meat. However, the achievable shelf-life of modified atmosphere packed raw poultry products depend on the species, fat content, initial microbial load, packaging type, gas mixture, and temperature of storage (Stiles, 1991). Holck et al. (2014) stored chicken fillets in 0, 30, 60, or 100% CO_2_ with N_2_ makeup gas and reported that the inhibition of microbial growth on raw chicken fillets was proportional to the CO_2_ concentration in package headspace. Sante et al. (1994) reported that poultry packed in a 100% CO_2_ atmosphere showed the lowest microbial counts in comparison to 100% O_2_, 100% N_2_, or a mixture of 25% CO_2_, 66% O_2_, and 9% N_2_. High CO_2_ packages also inhibit *Salmonella* effectively. Sukumaran et al. (2015) found that storage of untreated chicken breast fillets under MAP condition (95% CO_2_ and 5% O_2_) reduced *Salmonella* counts by 0.4 to 0.6 log10 CFU/g. Inhibition of *Salmonella* was noted for chicken legs stored in high barrier bags at 10°C under 100% CO_2_ but not for those in vacuum packages (Gray et al., 1984). In Europe, the typical MAP used by manufacturers for broiler meat contains 80% CO_2_ and 20% N_2_ (Susiluoto et al., 2003).

Plasma is a fundamental state of matter consisting of a gas of ions. Cold plasma (CP) is a plasma that is not in thermodynamic equilibrium and stays at temperatures well below those used for pasteurization or sterilization. Therefore, it is also called nonthermal plasma. During CP generation, gas molecules are broken down and form various reactive species (RGS), such as photos (UV), free radicals, energetic ions, and high oxidative atoms/molecules, which inactivate contaminating microbes on foods (Gaunt, 2006; Niemira, 2012). It has been well documented that the type of working gas employed principally determines the nature and quantities of RGS produced in the discharge as well as the efficacy of the treatment against microbes (Liao et al., 2019; Lopez et al., 2019). Rowan et al. (2007) investigated the effects of 4 working gases N_2_, CO_2_, O_2_, and air on the effectiveness of a CP (high-voltage pulsed-plasma gas-discharge) system to decontaminate poultry wash water. They found that the use of O_2_ alone produced the greatest reduction of *Bacillus cereus* endospores, followed by CO_2_, air, and N_2_. With a diffuse coplanar surface barrier discharge system, Hertwig et al. (2017) also evaluated N_2_, CO_2_, O_2_, and air for the efficacy of CP inactivation. They found that similar to O_2_ working gas, CO_2_ resulted in significant ozone formation (2,150 ppm) during discharge and inactivated *B. subtilis* wildtype spores. The D-value was 1.21 for air-plasma, 1.52 min for O_2_-plasma; however, it was 1.10 min for CO_2_. Takamatsu et al. (2015) used a multigas plasma jet to generate atmospheric nonthermal plasmas with various gases to treat microbial suspensions. Results showed that either CO_2_ or N_2_ plasma resulted in >6 log kill of *S. aureus* bacteria within 60 s, while O_2_ plasma produced a 3-log kill and air and Ar plasmas <1-log kill at 120 s. These results demonstrated that the utilization of CO_2_ as a working gas can effectively generate RGS in plasma and may enhance microbial inactivation by CP treatment.

In-package cold plasma (**IPCP**) is a type of nonthermal plasma system (Misra et al., 2019). In the IPCP system, plasma is generated with dielectric barrier discharge (**DBD**) and localized inside a sealed package to disinfect packed food materials. Compared with the other CP systems, the advantages of IPCP include reducing the risk of microbial contamination on packaging materials and postprocessing food and basically having no limits to food package configurations, materials, and atmosphere (Misra et al., 2019; Ziuzina et al., 2016). Experiments showed that effectiveness of the inactivation by the IPCP is also significantly influenced by working gases within packages or modified atmosphere. The modified atmosphere consisting of 30% CO_2_/65% O_2_ /5% N_2_ resulted in significant increases in the formation of RGS and inactivation of *B.* spores, spoilage bacteria, and pathogens on fresh poultry meat (Keener et al., 2012; Kronn et al., 2015; Zhuang et al., 2020). Therefore, the objective of the present study was to explore the interaction effects between high CO_2_ packaging and IPCP treatments on the quality and safety of raw chicken breast meat. The data collected from this study will provide useful information on the application of CP treatments in the retention of meat products.

## MATERIALS AND METHODS

### Meat Samples, Packaging, and Storage

Boneless skinless chicken breast fillets (*Pectoralis major*) were collected from a local commercial processing facility. A total of 100 cutlets and 60 whole fillets were collected for each trial. A total of 2 trials were conducted on separate dates using raw meat from different flocks. Prior to treatments, meat samples, containing 2 fillet cutlets (approximately 96.3 g each) and 2 muscle samples (2.5 cm diameter, 2.5–3.0 cm thick, average weight 21 g) cored from the cranial end of a single whole fillet (approximately 180.1 g), were placed in polymeric trays (19.5 cm x 14.5 cm x 4.0 cm, Sealed Air Corp., Duncan, SC). One cutlet was skin/ventral side up and used for microbial analysis (psychrophiles). The other cutlet was bone/dorsal side up and used for surface color (International Commission on Illumination [**CIE**] L*a*b*) measurements. One of the muscle core samples was inoculated with *Campylobacter* and the other with *Salmonella* with the inoculated side up. Individual trays were flushed with CO_2_ with make-up N_2_ gas for 0, 30, 70, or 100% of CO_2_ packages using a gas mixer (Gas Mixer KM-Flow, Witt-Gasetechnik, Deutschland, Germany), and sealed with a polypropylene-based barrier film (Toplex HB60, Plastopil Europe, Netherlands) using a tray sealer (Koch Kats 100 Single Head Tray Sealer, Ultra Source LLC, Kansas City, Missouri). Immediately after sealing, headspace compositions in packages were verified with a headspace gas analyzer (CheckPoint-Handheld Gas Analyzer, Dansensor, DK-4100 Ringsted, Denmark). The packaged samples were then set at ambient temperature for at least 45 min so that the relative humidity in the packages reached > 80% (based on a preliminary test result with a gas/humidity logger) before IPCP treatments. Packaged samples were then stored in a 4°C cold room for 5 d after IPCP treatments.

### Inoculation With Foodborne Pathogens Salmonella and Campylobacter

The microbiological protocols (including both inoculation and microbial analysis) used in this study were previously described in Rothrock et al. (2017) and Zhuang et al. (2019a). In short, *Campylobacter Jejuni* isolate and *Salmonella* Typhimurium isolate (both originally recovered from poultry carcasses rinses from a commercial poultry processing facility) were used during this study. The C. *jejuni* isolate was grown in Tryptic Soy Broth/Agar biphasic cultures, which were sealed in 2 gal Ziploc bags under under a hydrogen-enriched micro-aerobic atmosphere (7.5% H_2_, 2.5% O_2_, 10% CO_2_, and 80% N_2_) and incubated at 42°C, for 24 h (Lynch, Cagney, McDowell, & Duffy, 2010; Stintzi, 2003). The *S.* Typhimurium isolate was grown in Tryptic Soy Broth (TSB) at 37°C for 24 h at 200 rpm (Garrity, Bell, & Lilburn, 2005). After incubation, 5 mL of the *C. jejuni* or *S.* Typhimurium growth was added to 495 mL of 1 X phosphate buffered saline (**PBS**) to create the 10^6^ cfu/mL inoculum, which was verified spectrophotometrically (OD600) based on a standard curve (we were targeting a specific OD600 range. This allowed us to perform the inoculation with the isolates, but also then quantify the isolates using serial dilution plating to have a specific starting concentration from which to determine the log reduction of the treatments). The actual concentrations of inocula were 6.6 ± 0.3 log_10_ for *Campylobacter* and 6.9 ± 0.4 log_10_ for *Salmonella*. The inoculation process began by pipetting 75 mL of inoculum into a plastic food tray (CS979, Cryovac, Duncan, SC). Fillet core samples were placed skin side down in each tray so that only the surface sat in the liquid. The core samples soaked for 30 min to allow the microorganisms to attach to the surface. After soaking, the core samples were removed and allowed to drip for 5 min before they were placed in the tray package (Kronn et al., 2015).

### In-Package DBD-CP Treatment

The same IPCP device as described by Kronn et al. (2015) was used in this study. The treatments were performed with a BK-130 AC dielectric test set consisting of a high voltage transformer, power supply, and control system (Phenix Technologies, Accident, Md.). Two 15.24 cm diameter spun-aluminum electrodes were connected to the high-voltage transformer. The 2 electrodes were arranged parallel to one another on the top and bottom of the sample. The electrodes were separated from the sample package by dielectric barriers and insulated from the bench with a yellow low-voltage electrical blanket (Velcro, 36 × 36 in, Class 0 Type 1, Salisbury standard rubber insulating blanket, Lab Safety Supply, Chicago, Ill.). The top electrode was connected to the 130 kV tap of the high-voltage transformer with a 1.09 m high-voltage spark plug wire (8.5 mm super conductor spark plug wire, MSD, El Paso, Tex.). For additional insulation, the high-voltage wire was fed through a 1.09 m length of Tygon tubing of 1.27 cm diameter. The bottom electrode was connected to the return terminal of the transformer with the supplied ground wire, and a jumper connected the ground and guard terminals. Before treatments, the system was warmed up with a sealed empty tray at 80 kV for at least 5 min. Sample packages were treated at 70 kV for 3 min. To ensure treatment consistency, meat package temperature was checked before and after treatment and treatment voltage and current were monitored during DBD. The plasma power or total energy was 55.7 ± 7.3 Watts. The average temperature of meat packages prior to treatments was 23.0 ± 0.7°C and the average temperature of meat packages immediately after treatments was 32.5 ± 2.2°C.

### Ozone Gas Measurement

Ozone gas was used as a reference for system performance, as it served as an indicator for cold plasma generation and antimicrobial activity within the package (Klockow and Keener, 2009; Kronn et al., 2015; Wang et al., 2016; Wang et al., 2017; Ziuzina et al., 2013; Ziuzina et al., 2014). Immediately following IPCP treatments, headspace gas was taken from the sample tray and ozone content in the package was measured using a spectral method. A gas-tight syringe (25 mL Gastight #1025, Hemilton Co., Reno, Nevada) was used to collect gas samples (10 mL) from the package headspace. The syringe volume (with the needle on) was expelled into a 0.2 cm UV cell, which was inserted in a Jenway 7305 Spectrophotometer (Bibby Scientific, Ltd, Stone, Staffs, UK), through tubing that was connected to cell inlet. Absorbance values were recorded. Ozone concentration was calculated using the following formula based on the Beer-Lambert law: A = Ɛ x l x c, where Ɛ is the extinction coefficient of ozone gas at 254 nm, l is the cell path length, and c is the ozone concentration in mol L^−1^ (**M**). An Ɛ value of 3000 M^−1^cm^−1^ was used (Rakness, et al., 1996). Resultant concentration values were converted to parts per million by volume (**PPMV**) using the ideal gas equation. The UV cell was flushed with air (at least 10 mL) between ozone measurements.

### Microbial Analysis

For microbiological recovery, the fillet cutlets or cores (including 3 extra cutlets and 3 meat cores inoculated with *C. Jejuni* and 3 meat cores inoculated with *S.* Typhimurium for initial microbial load and the samples treated with IPCP and stored for 5 d) were rinsed with phosphate buffered saline (PBS, Gibco by Life Technologies, Grand Island, NY), about 22.5 mL for a cutlet and 5.25 mL for a cylinder. The PBS diluents were then serially diluted 4 times (∼10^7^ to 10^3^ CFU/mL). *C. jejuni* rinse dilutions were plated onto Campy-Cefex agar. The *Salmonella* was initially recovered using TSB from frozen stocks. This *Salmonella* isolate possessed resistance to nalidixic acid (NAL). So in order to recover only the *Salmonella* isolate we inoculated the fillet with (and not any potential naturally occurring already on the fillet), the Brilliant Green Sulfa agar (**BGS**) + 100 ppm NAL was used. This was the same method used in the previously published paper (Zhuang et al., 2019a; Zhuang et al. 2019b; Zhuang et al., 2020) to evaluate log reductions based on these types of treatments. Both *Campylobacter* and *Salmonella* were incubated at the same temperature and atmosphere conditions as described in section 2.2. To assess the effect of the treatment on naturally occurring psychrophiles populations (representative of spoilage organisms), rinse dilutions were plated onto TSA agar and incubated at 4°C for 10 d. Plates containing between 30 and 300 colonies were used for enumeration of *Salmonella, Campylobacter*, and psychrophile. Since selective and differential plates were used for both *Salmonella* and *Campylobacter*, only the colonies that displayed the appropriate colony characteristics were counted.

### Drip Loss, pH, and Surface Color Measurements

For drip loss, chicken cutlets were weighed before they were packed in the trays and after removal from storage. Drip loss was calculated by the formula below:

$$\text{Drip}\text{loss}\left(\%\right)=100\times \left[\left(W_{\text{initial}}-W_{\text{post}-\text{storage}}\right)/W_{\text{initial}}\right]$$

where W_initial_ was cutlet weight measured prepackaging; W_post-storage_ was cutlet weight measured after the samples were stored at 4°C for 5 d.

Surface pH of chicken fillets was measured using a pH meter (Extech pH100, LIR Systems, Inc., Nahua, NH) with a flat top probe placed perpendicular to the surface of the meat. Measurements were carried out before samples were sealed in the package and after storage. pH changes were expressed as pH_post-storage_ – pH_initial_; where pH_initial_ was measured prepackaging; pH_post-storage_ was measured after the samples were stored at 4°C for 5 d.

The surface color (CIE L*a*b*) on the bone-side surface of the chicken cutlets was measured using a spectrophotometer (CM-2600 d, Konica Minolta, Inc., Tokyo, Japan) with settings of illuminant C, 10° observer, specular component excluded, and an 8-mm aperture. Color was measured before fillets were packed and after the samples were treated and stored at 4°C for 5 d. Surface areas free from obvious defects (bruises, discolorations, hemorrhages, or any other conditions that might have prevented uniform color readings) were selected for measurements. Two measurements were taken for each fillet cutlet. Results were expressed as differences in L*a*b* values between initial measurements before fillets were packed and final measurements after 5 d of storage.

### Statistical Analysis

A factorial design was used for the experiments. The entire experiment was independently replicated 2 times. Microbial and quality measurements in each experiment were performed in triplicate (6 pieces of cutlets per treatment). Data analyses were performed using 2-way ANOVA via the Proc GLMMIX of SAS. Cold plasma treatment and CO_2_ levels were the main effects and replication was a random effect. Means were separated using Tukey's range test (*P* < 0.05). For pH and color, the differences in parameters between poststorage and pretreatment (fresh meat) were calculated first and then used for the ANOVA analysis.

## RESULTS AND DISCUSSION

### Ozone Formation in Package

Table 1 shows the effect of IPCP treatments on ozone formation in packages with elevated CO_2_ content. Virtually no ozone formation was detected in 0, 30, and 70% CO_2_ packages after packages were treated at 70 kV for 3 min (*P* > 0.05); however, significant ozone formation (21.9 ppm) was observed in 100% CO_2_ packages after CP treatments (*P* < 0.05). With CO_2_ as the sole working gas, Hertwig et al. (2017) measured ozone formation during a diffuse coplanar surface barrier discharge. The ozone formation in 100% CO_2_ packages could be due to CO_2_ decomposition during DBD. Recently, several published reports have shown that nonthermal DBD-based plasma treatments can decompose CO_2_ (Mei et al., 2016a, b; Zeng and Tu, 2017; Ramakers et al., 2015; Wang et al., 2017; Mei and Tu, 2017). Ray and Subrahmanyam (2016) and Wang et al. (2017) demonstrated the formation of O_2_ and single O when 100% CO_2_ gas passed a DBD plasma reactor. Horváth et al. (2008) found CO and ozone were the main products in plasma-induced CO_2_ decomposition. In addition, Ray and Subrahmanyam (2016) also showed that dielectric materials in the DBD plasma reactor could affect CO_2_ decomposition. Our present study indicates that CO_2_ decomposition may also occur in raw meat packages during DBD-based CP treatments.

**Table 1:** Effects of dielectric barrier discharge on ozone formation in high CO_2_ chicken meat packages.

CO_2_ Content (%)*	Ozone formation (ppm)
0	0 ± 0^b^
30	0.5 ± 0.5^b^
70	0.0 ± 0.0^b^
100	21.9 ± 7.5^a^

a-b Means with no common letter differ significantly (*P* < 0.05).

⁎ Make-up gas in packages was N_2_.

### CO_2_ Contents in Packages

Table 2 shows the effects of the IPCP treatment on headspace gas CO_2_ content in raw chicken breast meat packages flushed with different levels of CO_2_ immediately after the backflushing (initial CO_2_) and after 5-d storage. The initial CO_2_ content in packages were consistent with the targeted concentrations for CO_2_ treatments (0.3% for 0%; 30.6% for 30%; 71.1% for 70%; and 100.0 for 100% targeted concentration) in our experimental design. Storage time and especially IPCP treatments affected CO_2_ content in packages (*P* < 0.05). In the elevated CO_2_ content (≥30%) packages, IPCP treatments with refrigerated storage resulted in reduced CO_2_ (*P* < 0.05). For example, CO_2_ content reduced more than 8% in 30% package; it was more than 20% in 70% package; and 13% in 100% CO_2_ packages (Table 2). There were also consistent reductions (more than 5%) in CO_2_ content in the elevated CO_2_ nontreated packages after storage compared with the initial content, although a statistical difference was only noticed for the 70% CO_2_ treatment (*P* < 0.05). A similar relationship was noted between the IPCP-treated and nontreated packages, with treated packages being consistently lower (more than 3%) than nontreated packages. The reduction in CO_2_ in modified atmosphere food packages has been attributed to high dissolution of CO_2_ in a food matrix (Chaix et al., 2014). These results also indicate that CP treatments may significantly impact CO_2_ content in the package. The reduction in CO_2_ content in the treated packages may result from decomposition of CO_2_ by DBD-based CP treatments (Zhang et al., 2017; Ray and Subrahmanyam, 2016; Ray et al., 2017). The reason why CO_2_ content in 70% treatment reduced much more (more than 10% in nontreated control and more than 20% in IPCP-treated package than the other treatments (less than 9% in 30% treatment and less than 15% in 100% treatment) is unknown. However, the standard error indicated that the results may not be solely due to the experimental variation (Table 2).

**Table 2:** Effects of cold plasma treatment and storage on CO_2_ content in packages.

CO_2_ treatment (%)*	Initial CO_2_ (%)	CO_2_ in nontreated package (5 d)	CO_2_ in treated package (70 kV for 3 min + 5 d)
0	0.3 ± 0.0^h^	3.4 ± 0.1^h^	3.3 ± 0.1^h^
30	30.6 ± 0.1^f^	25.1 ± 0.3^fg^	22.1 ± 0.9^g^
70	71.1 ± 0.7^c^	60.5 ± 0.9^d^	48.6 ± 2.3^e^
100	100.0 ± 0.1^a^	93.7 ± 2.6^ab^	87.0 ± 3.5^b^

a-h Means with no common letter differ significantly (*P* < 0.05).

⁎ Make-up gas in packages was N_2_.

### Microbiota Populations on Raw Chicken Fillets

Table 3 shows the effect of IPCP treatments on psychrophiles on raw chicken breast meat packed in high CO_2_ atmosphere. Statistical analysis showed that there was no interaction between CO_2_ content in package and IPCP treatment (*P* > 0.05). However, both CO_2_ and IPCP affected psychrophilic populations on raw chicken breast meat (*P* < 0.05). Cold plasma treatments resulted in more than a 60% (or 0.4 log10 cycles) reduction in overall mean of bacterial populations (from 7.00 to 6.57 log in overall mean of cold plasma treatment row). CO_2_ of 30% in package resulted in more than a 0.6 log10 cycles reduction (from 8.08 to 7.44 log in CO_2_ mean column); 70% and 100% CO_2_ resulted in more than a 1.4 log10 cycle reduction (from 8.08 to 6.63 and 6.35 log, respectively). These results further demonstrated that either IPCP treatments or high CO_2_ content in packages can effectively reduce spoilage microorganisms on raw poultry meat. High CO_2_ gas was more effective against psychrophilic growth than IPCP treatments.

**Table 3:** Effects of cold plasma treatment and storage on populations (Log 10 CFU/mL) of psychrophiles on raw chicken fillets packed in different CO_2_ atmospheres.

	Psychrophiles			Factor Significance		
CO_2_ Content (%)*	Control (nontreated)	Treatment (70 kV for 3 min)	CO_2_ mean	CO_2_	Cold plasma	CO_2_ x Cold plasma
— (0 d)	3.85	—	3.85^d^	< 0.001	0.033	0.648
0 (5 d)	8.09	8.08	8.08^a^	SEM (CO_2_) = 0.20		
30 (5 d)	7.59	7.30	7.44^b^	SEM (cold plasma) = 0.18		
70 (5 d)	6.84	6.41	6.63^c^	SEM (CO_2_ x cold plasma) = 0.23		
100 (5 d)	6.49	6.20	6.35^c^			
Overall mean of cold plasma treatments	7.00^a^	6.57^b^				

a-d Means with no common letter differ significantly (*P* < 0.05). SEM = the standard error of the mean.

⁎ Make-up gas in packages was N_2_.

There was no interaction effect between CO_2_ content and IPCP treatment or effect of CO_2_ content by itself (*P* > 0.05) on *Campylobacter Jejuni* populations on raw chicken breast meat packed in high CO_2_ atmosphere (Table 4). However, overall mean of *C. Jejuni* populations on IPCP-treated raw chicken breast meat (4.15 log) were more than 0.6 log10 cycles lower than overall mean on the nontreated controls (4.82) (*P* < 0.05). These results further demonstrate that enhanced CO_2_ content in anaerobic package does not affect *Campylobacter* populations on raw chicken meat; however, the IPCP treatment significantly reduced *Campylobacter* populations on raw chicken meat even in anaerobic/high CO_2_ packages.

**Table 4:** Effects of cold plasma treatment and storage on populations (Log 10 CFU/mL) of *Campylobacter Jejuni* on raw chicken fillets packed in different CO_2_ atmospheres.

CO_2_ Content (%)*	*C. jejuni* (log10 cfu/mL)			Factor Significance		
	Control (nontreated)	Treatment (70 kV for 3 min)	CO_2_ mean	CO_2_	Cold plasma	CO_2_ x Cold plasma
— (0 d)	4.98	—	4.98	0.163	<0.001	0.939
0 (5 d)	4.73	4.07	4.40	SEM (CO_2_) = 0.09		
30 (5 d)	4.64	4.03	4.34	SEM (cold plasma) = 0.06		
70 (5 d)	4.77	4.23	4.50	SEM (CO_2_ x cold plasma) = 0.13		
100 (5 d)	4.97	4.27	4.62			
Overall Mean of Cold Plasma Treatments	4.82^a^	4.15^b^				

Note: *C.* Typhimurium population used for the inoculation was log_10_ 6.61 CFU/mL.

a-b Means with no common letter differ significantly (*P* < 0.05). SEM = the standard error of the mean.

⁎ Make-up gas in packages was N_2_.

There was no interaction effect between CO_2_ content and IPCP treatment and no difference between the nontreated and IPCP treated samples (*P* > 0.05) regarding *S.* Typhimurium on raw chicken breast meat packed in high CO_2_ atmosphere (Table 5). However, 100% CO_2_ content in packages reduced *S.* Typhimurium populations (*P* < 0.05) on raw chicken breast meat (more than 0.5 log10 cycles from 4.94 to 4.39 log in CO_2_ mean column), although no difference (*P* > 0.05) was noted between 0% CO_2_, 30, and 70% CO_2_. These results demonstrate that IPCP treatment does not influence *Salmonella* populations on raw chicken meat in anaerobic/high-CO_2_ packages. Increasing CO_2_ content in anaerobic packages can reduce *S*. Typhimurium population on raw chicken meat.

**Table 5:** Effects of cold plasma treatment and storage on populations (Log 10 CFU/mL) of *Salmonella* Typhimurium on raw chicken fillets packed in different CO_2_ atmospheres.

	*S.* Typhimurium (log10 cfu/mL)			Factor Significance		
CO_2_ Content (%)*	Control (nontreated)	Treatment (70 kV for 3 min)	CO_2_ mean	CO_2_	Cold plasma	CO_2_ x Cold plasma
— (0 d)	4.85	—	4.85^ab^	0.004	0.206	0.427
0 (5 d)	4.73	5.15	4.94^a^	SEM (CO_2_) = 0.42		
30 (5 d)	4.86	5.07	4.97^a^	SEM (cold plasma) = 0.41		
70 (5 d)	4.66	4.66	4.66^ab^	SEM (CO_2_ x cold plasma) = 0.43		
100 (5 d)	4.42	4.37	4.39^b^			
Overall Mean of Cold Plasma Treatments	4.81	4.70				

Note: *S.* Typhimurium population used for the inoculation was log_10_ 6.43 CFU/mL.

a-b Means with no common letter differ significantly (*P* < 0.05). SEM = the standard error of the mean.

⁎ Make-up gas in packages was N_2_.

Inactivation of microbes by IPCP treatments in high CO_2_ experiments agrees with previous reports. Kronn et al. (2013); Kronn et al. (2015) demonstrated that the same DBD-based IPCP treatment resulted in about 2.0 log10 CFU/mL reductions in both spoilage organism and *C. Jejuni* on chicken breast meat packed in a modified atmosphere (65% O_2_ + 30% CO_2_) after storage. Rothrock et al. (2017) evaluated the same IPCP system for inactivating microbial organisms *Pseudomonas, Campylobacter*, and *Salmonella* in suspensions and found that *Campylobacter* bacterium was much more sensitive to IPCP treatments than *Salmonella* and *Pseudomonas*.

It has been well noted that high CO_2_ in packages can significantly extend microbiological shelf life of poultry meat (Stiles, 1991). Jimenez et al. (1997) packaged chicken breasts in air, under vacuum, or high CO_2_ (30% or 70% CO_2_ with N_2_ as a make-up gas) modified atmosphere and found that spoilage bacteria pseudomonas grew well in air or under vacuum, but growth was suppressed in the high CO_2_ modified atmosphere packaging. Sante et al. (1994) reported that poultry packed in a 100% CO_2_ atmosphere showed the lowest microbial counts in comparison to 100% O_2_, 100% N_2_, or a mixture of 25% CO_2_, 66% O_2_, and 9% N_2_. Holck et al. (2014) stored chicken fillets in 0, 30, 60, or 100% CO_2_ with makeup gas N_2_ and reported that the inhibition of microbial growth on raw chicken fillets was proportional to the CO_2_ concentration in package headspace. For *Salmonella*, inhibitory effect by high CO_2_ in packages has also been observed. Sukumaran et al. (2015) found that storage of untreated chicken breast fillets under MAP condition (95% CO_2_ and 5% O_2_) reduced *Salmonella* counts by 0.4 to 0.6 log10 CFU/g. Baker et al. (1986) reported around 0.5 log10 CFU/g reduction of *S.* Typhimurium in ground chicken when stored under 80% CO_2_. Al-Haddad et al. (2005) reported reduction (< 1 log) in *S. Infantis* on chicken skin stored under 70% CO_2_/30% N_2_ conditions. Inhibition of *Salmonella* was noted for chicken legs stored in high barrier bags at 10°C under 100% CO_2_ but not for those in vacuum packages (Gray et al 1984). On the other hand, Wesley et al. (1985) studied the behavior of *Campylobacter Jejuni* at 4°C on broilers in oxygen permeable packs and in packs containing 100% CO_2_ and found that CO_2_-enriched atmosphere had no detectable effect on the organism. With turkey rolls, Phebus et al. (1991) reported that increasing concentrations of CO_2_ inside the packs from 0 to 100% resulted in lower rates of inactivation of *Campylobacter* regardless of storage temperature. Meredith et al. (2014) concluded that CO_2_ in the MAP mixture apparently assisted the survival of *Campylobacter* and attributed the survival to the CO_2_ inhibition of other microorganisms that compete with *Campylobacter* in air.

### Quality Indicators of Raw Chicken Fillets

Table 6, Table 7, Table 8 show the changes in pH and color L*a*b* before and after IPCP treatment and storage. The reason for using the changes in these quality measurements for IPCP and CO_2_ effects is to avoid the effects on measured results due to variation in raw chicken meat. The variation in color and pH of chicken breast meat has been demonstrated in many published reports (Fletcher, 1999; Petracci et al., 2009).

**Table 6:** Effects of cold plasma treatment on pH changes in raw chicken fillets packed in different high CO_2_ atmospheres.

CO_2_ Treatment (%)	pH change* in nontreated fillet (5 d)	pH change in Treated fillets (70 kV for 3 min + 5 d)
0	-0.10 ± 0.16^a^	0.01 ± 0.08^ab^
30	-0.19 ± 0.02^a^	0.18 ± 0.09^a^
70	-0.09 ± 0.03^a^	-0.17 ± 0.00^ab^
100	-0.28 ± 0.08^ab^	-0.31 ± 0.03^b^

Notes: Mean ± stderr; P-value (CO_2_) = 0.009; P-value (cold plasma) = 0.114; P-value (CO_2_ x cold plasma) = 0.046.

a,b Means with no common letter differ significantly (*P* < 0.05).

⁎ pH changes = pH_post-storage_ – pH_initial_.

There was no interaction (*P* > 0.05) between IPCP treatment and package CO_2_ content for drip loss. There were no differences (*P* > 0.05) in drip loss between the treatments regardless of IPCP or CO_2_ content in packages (data not shown). However, interaction between IPCP treatment and CO_2_ content existed (*P* < 0.05) for pH changes in samples (Table 6). There were no differences in meat pH changes between nontreated and treated samples regardless of CO_2_ content in packages (*P* > 0.05). Changes in pH (absolute value) were consistently greater in 100% CO_2_ packages than in 0% CO_2_ packages. It was more than 0.15 units in untreated fillets and mor 0.30 units differences in IPCP treated fillets (Table 6). These data indicate that CO_2_ content could affect pH of chicken breast meat in packages under our experimental conditions. Negative value of pH changes indicated a decrease in pH on surface of meat samples after storage. Overall, these data are consistent with published reports. Zhuang et al. (2020) and Wang et al. (2017) found no difference in meat pH between nontreated and plasma-treated samples after storage. Although CO_2_ in the packaging atmosphere could theoretically be absorbed in the food, resulting in the formation of H_2_CO_3_ and subsequently reduced pH in the food matrix, the effects of high CO_2_ package on meat pH have not been consistent in published reports. For example, Jakobsen and Bertelsen (2002) reported that a decrease in pH of 0.05 to 0.35 units was observed in 5 out of fifteen studies on red meat packed in elevated CO_2_ atmosphere. With poultry meat, Hotchkiss et al. (1985) studied high CO_2_ (up to 80%) for packaging poultry and found no clear effect on pH of chicken quarters due to elevated CO_2_. Baker et al. (1985) stored ground chicken meat under elevated CO_2_ (up to 100%) and found that the differences in pH was within 0.10 units throughout the test period. Chouliara et al. (2007); Chouliara et al. (2008) and Patsias et al. (2006) did not observe significant effects of high CO_2_ packaging on pH in chicken breast meat. However, Jurdi et al. (1980) found that the pH values of mechanically deboned chicken meat treated with elevated CO_2_ (up to 100%) were consistently lower compared with those samples exposed to 100% N_2_ or air regardless of fat content. The controversy has been attributed to buffering capacity of the meat stored in high CO_2_ atmospheres (Jakobsen and Bertelsen, 2002).

Table 7 shows the effect of IPCP treatments on differences in surface color CIE L* values of raw chicken breast meat packed with different CO_2_ contents. Statistical analysis showed no interaction effect (*P* > 0.05); however, both CO_2_ and CP affected L* values (*P* < 0.05). The change in L* values between CP treated and stored samples and pretreatment samples was greater (*P* < 0.05) than for the nontreated samples (more than 1.5 units), indicating that CP treatment resulted in significant increases in lightness of raw chicken breast meat. High CO_2_ content in packages also resulted in increasing (*P* < 0.05) changes in L* value (from -0.82 to 3.53) and there were no differences in L* value changes (*P* > 0.05) in treated meat after storage between the 3 high CO_2_ packages. These results indicate that higher CO_2_ levels may cause increased lightness in raw chicken meat.

**Table 7:** Effects of cold plasma treatment on changes in L* values (lightness) of raw chicken fillets packed in different high CO_2_ atmospheres.

CO_2_ Content (%)	ΔL* (= L*_post-storage_ – L*_initial_)			Factor Significance		
	Nontreated	Treated (70 kV for 3 min)	CO_2_ mean	CO_2_	Cold plasma	CO_2_ x Cold plasma
0	−2.41	0.77	−0.82^b^	P < 0.001	P = 0.016	P = 0.125
30	1.79	0.96	1.37^ab^	SEM (CO_2_) = 0.84		
70	1.43	2.70	2.07^a^	SEM (cold plasma) = 0.69		
100	2.00	5.05	3.53^a^	SEM (CO_2_ x cold plasma) = 0.125		
Overall Mean of Cold Plasma Treatments	0.70^b^	2.37^a^				

a-b Means with no common letter differ significantly (*P* < 0.05).

Table 8 shows the effect of IPCP treatments on differences in surface color CIE a* and b* values of raw chicken breast meat packed with different CO_2_ contents. Statistical analysis showed no interaction effect (*P* > 0.05) between CO_2_ content and CP treatment and CO_2_ content by itself on changes in a* and b* values. However, CP affected the changes in both a* and b* values (*P* < 0.05). Cold plasma treatments increased changes in a* by more than 0.5 units and decreased changes in b* by more than 0.9 units. These results indicate that CP treatment could cause increases in redness and decreases in yellowness of raw chicken breast meat after refrigerated storage.

**Table 8:** Effects of cold plasma treatment on changes in a* (redness) and b* (yellowness) of raw chicken fillets packed in different high CO_2_ atmospheres.

CO2 content (%)	Δa* (= a*_post-storage_ – a*_initial_)			Δb* (= b*_post-storage_ – b*_initial_)		
	Nontreated	Treated + 5d storage		Nontreated		Treated + 5d storage
0	0.63	1.47		−0.77		−0.05
30	0.72	1.50		0.89		−0.51
70	0.79	1.07		1.00		−0.59
100	0.80	1.13		0.72		−0.64
Overall Mean of Cold Plasma Treatments	0.73b	1.29^a^		0.46^a^		−0.45^b^
Significance for a*	P (CO_2_) = 0.675		P (cold plasma) = < 0.001		P (CO_2_ x cold plasma) = 0.193	
Significance for b*	P (CO_2_) = 0.582		P (cold plasma) = 0.015		P (CO_2_ x cold plasma) = 0.858	

a-b Means with no common letter within a parameter differ significantly (*P* < 0.05).

Similar findings have been reported in published literature. For example, with the same IPCP system, Zhuang et al. (2019a); Zhuang et al. (2019b) showed that IPCP treatments resulted in increased L* values. Wang et al. (2017) found IPCP treatment could significantly affect a* and b* values of raw chicken breast fillets. Zhuang et al. (2020) reported that IPCP treatment significantly affected a* and b* values of raw chicken breast meat and the effects varied with IPCP treatment conditions, such as voltage, treatment time, and package headspace. Baker et al. (1985) and Jurdi et al. (1980) reported that high CO_2_ atmospheres resulted in increased L* value of further processed chicken products. Hotchkiss et al. (1985) demonstrated that CO_2_-stored samples had lower scores for color of chicken quarters. In addition, Patsias et al. (2006) and Chouliara et al. (2007, 2008) found that elevated CO_2_ package had no effects on a* and b* values of raw chicken breast meat. Our present study demonstrated that either high CO_2_ package or cold plasma treatments could result in changes in the appearance of raw chicken breast meat.

## CONCLUSIONS

Our data showed that there were no interaction effects between CO_2_ and in-package cold plasma treatment for most quality parameters except for changes in meat pH and CO_2_ content in packages under the conditions evaluated in this study. The cold plasma treatment significantly reduced spoilage microbial growth and *Campylobacter* populations on the meat surface but did not affect *Salmonella* populations regardless of CO_2_ content in packages. Increasing CO_2_ in packages significantly reduced spoilage microbial growth and *Salmonella* populations regardless of cold plasma treatment and the effect was greater than that of the cold plasma treatment. However, CO_2_ did not affect *Campylobacter* populations. The cold plasma treatment affected color parameters, causing changes in lightness, redness, and yellowness (increased L* and a* values and reduce b* values, respectively) on the surface of treated raw chicken breast meat. These results indicate that it affects appearance of raw chicken meat. High CO_2_ in packages (30 to 100%) also caused increases in lightness (L* values) of the raw chicken meat. However, further increases in CO_2_ above 30% did not significantly increase changes in meat L* values. The recommended treatment conditions for extending microbiological shelf life and reducing foodborne pathogens on fresh chicken breast meat in higher CO_2_ packages for DBD-based in-package cold plasma treatments is 100% CO_2_ and 70 kV for 3 min. Further treatment parameter optimization is needed to reduce the adverse effects of high CO_2_ package and cold plasma treatment on appearance of treated poultry breast meat as well as further enhance the antimicrobial effectiveness against foodborne pathogens.

## DISCLOSURES

The authors have no conflicts of interest to declare.
